# A predictive model for hip abductor strength and knee extensor strength 12 months after total hip arthroplasty with an interaction term

**DOI:** 10.1186/s12891-021-04719-2

**Published:** 2021-09-27

**Authors:** Junya Sekita, Naonobu Takahira, Genki Iwamura, Hiroyuki Watanabe, Atsushi Kusaba, Saiji Kondo

**Affiliations:** 1 Graduate School of Medical Sciences, Kitasato University, 1-15-1 Kitasato, Minami-ku, Sagamihara-shi, Kanagawa 252-0373 Japan; 2 Department of Rehabilitation, Zama General Hospital, 1-50-1 Soubudai, Zama-shi, Kanagawa 252-0011 Japan; 3 Department of Orthopaedic Surgery, Kitasato University Graduate School of Medical Sciences, 1-15-1 Kitasato, Minami-ku, Sagamihara-shi, Kanagawa 252-0373 Japan; 4 Department of Rehabilitation, School of Allied Health Sciences, Kitasato University, 1-15-1 Kitasato, Minami-ku, Sagamihara-shi, Kanagawa 252-0373 Japan; 5 Institute of Joint Replacement and Rheumatology, Zama General Hospital, 1-50-1 Soubudai, Zama-shi, Kanagawa 252-0011 Japan

## Abstract

**Background:**

Identifying populations with poor muscle recovery after total hip arthroplasty (THA) is important for postoperative physical therapy. Preoperative muscle strength is a strong factor that determines postoperative muscle strength. However, this effect may depend on other factors. Thus, predictive models with interaction terms are important for accurately predicting postoperative muscle strength. This study aimed to develop a predictive model for lower muscle strength 12 months after THA which incorporates interaction terms.

**Methods:**

Subjects were female patients with hip osteoarthritis who underwent unilateral THA. Patients with locomotor disorders, neurological disorders, or postoperative complications were excluded. Hip abductor and knee extensor strength were measured, and a generalized linear model approach with preoperative muscle strength, age, body weight, height, disease duration, physical activity, and leg extension as explanatory variables was used to identify factors that determine muscle strength 12 months after THA. Models with interaction terms between preoperative muscle strength and other explanatory variables were also examined.

**Results:**

A total of 82 patients were analyzed. Preoperative muscle strength, age, body weight, physical activity, and disease duration were extracted as factors that significantly and independently determine hip abductor and knee extensor strength. The interaction term between preoperative muscle strength and age was identified as a factor that significantly determines knee extensor strength. Regression coefficients for preoperative knee extensor strength and postoperative muscle strength were significant when age was +1 SD, but not when age was -1 SD.

**Conclusions:**

The predictive model demonstrated that lower muscle strength 12 months after THA is determined by preoperative muscle strength, age, weight, physical activity, disease duration, and preoperative muscle strength, with the effect of preoperative muscle strength on knee extensor strength being dependent on age. When predicting postoperative knee extensor strength using preoperative muscle strength, it is important to consider the effect of age.

## Background

Total hip arthroplasty (THA) is an effective treatment for reducing hip pain and restoring hip joint mobility [1]. Many patients achieve pain reduction and improvements in activities of daily living and health-related quality of life after THA [2–4]. On the other hand, postoperative walking ability and muscle strength around the hip joint on the operated side may not reach the level of healthy subjects even 12 months after surgery [5–7]. Similarly, knee extensor strength on the operated side may not recover to the level of healthy subjects 6-12 months after surgery [6, 8]. Therefore, in terms of muscle strength at 12 months post-THA, recovery of not only hip abductor strength but also knee extensor strength remains an issue. Lower limb muscle strength affects gait function [9, 10], and is an important factor associated with the performance of activities of daily living [11]. Insufficient recovery of hip abductor and knee extensor strength following THA can lead to serious problems, such as falls [12, 13]. Thus, it is crucial to identify patients with poor muscle strength recovery and target these patients for intensive training prior to or early after surgery. The ability to accurately predict postoperative muscle recovery could contribute to these efforts.

Postoperative physical function and muscle strength are determined by a number of factors including age, sex, body mass index, disease duration, and surgical method [14–18]. In particular, preoperative physical function and muscle strength have been reported to be the most influential factors [15, 16]. In addition, postoperative physical activity, leg length, and disease duration are also closely related to muscle strength and may serve as factors which independently determine postoperative muscle strength. The effect of preoperative muscle strength on postoperative muscle strength may also depend on these factors. In terms of age, low preoperative muscle strength may have a strong effect on elderly people and a weak effect on younger populations. On this basis, we hypothesized that, while elderly people would not show sufficient recovery of muscle strength 12 months after surgery, young populations may achieve good recovery regardless of preoperative muscle strength. Similar tendencies may also exist for other factors, highlighting the importance of considering interaction terms between preoperative muscle strength and each factor to create a highly accurate predictive model.

The present study aimed to develop a predictive model for lower muscle strength 12 months after THA with an interaction term.

## Materials and methods

### Subjects

Subjects were female patients with hip osteoarthritis who underwent THA at two hospitals (Ebina General Hospital and Zama General Hospital) between December 2015 and September 2017. Inclusion criteria were patients who (1) underwent primary unilateral cementless THA via a minimally invasive anterolateral approach; (2) were female; and (3) agreed with the purpose of the study. Subjects were limited to female patients in order to minimize the effects of gender differences in lifestyle, as roughly 90% of our patients are female. Exclusion criteria were (1) a history of lower limb or spine surgery; (2) previously diagnosed painful orthopedic disease other than hip joint disease; (3) previously diagnosed end-stage hip osteoarthritis of the non-surgical side; (4) previously diagnosed mental disease or neuromuscular disease; and (5) postoperative complications such as fracture, dislocation, infection, or nerve paralysis. For comparison, 50 healthy community-dwelling women (age range, 45-78 years) who did not have lower limb joint pain were selected as controls.

Four highly-experienced surgeons performed all THAs without a navigation system. All THAs were performed in the half lateral position (i.e., pelvis tilted 60 degrees relative to the floor) through a minimally invasive anterolateral approach independent of the implant type [19, 20]. After an incision was made to the fascia lata at 0.5 mm posteriorly to the posterior border of the tensor fasciae latae muscle, the joint was approached between the gluteus medius muscle and the tensor fasciae latae muscle without damaging the muscles. The anterior iliofemoral ligaments, the anterior part of the capsule, and the conjoined tendon were preserved. We created a hip center similar to the anatomical hip center by acetabular reaming up to the lamina interna [21], as not doing so would result in the hip center being located superior-laterally to the anatomical hip center in the dysplastic acetabulum. By creating a hip center in its accurate anatomical position, the muscles can function normally [21, 22]. All implants were cementless. For stems, the SL-Plus stem (Smith & Nephew), Short Modular Femoral Hip System (Smith & Nephew), and Global Tissue Sparing stem (Zimmer Biomet) were used in 74.4%, 12.2%, and 7.8% of cases, respectively. For acetabular components, the R3 Acetabular System (Smith & Nephew), Continuum Acetabular System (Zimmer Biomet), and G7 Acetabular System (Zimmer Biomet) were used in 57.8%, 30.0%, and 7.8% of cases, respectively. Ceramic-on-ceramic bearings (Biolox Delta, Ceramtec AG) were used in 90% of patients, and ceramic-on-highly cross-linked polyethylene bearings (Biolox Delta, Ceramtec AG or Oxiniuum, Smith & Nephew) were used for the remaining 10%. These components were selected based on patient age and X-ray images of the hip joint.

This study complied with the ethical standards of the Declaration of Helsinki (1964) and its subsequent amendments and was approved by the Ethics Review Committees of Ebina General Hospital and Zama General Hospital (No. 207). Each patient provided written informed consent to participate in this study.

### Role of funding source

This study was supported by research funds from our institution.

### Procedures

This study was a 12-month prospective observational study. Postoperative rehabilitation was performed according to the clinical pathway of our institution. On the day after surgery, all patients were allowed to bear full weight and underwent inpatient rehabilitation. Rehabilitation consisted of gait exercises, passive range of motion exercises, and muscle strengthening exercises. For gait exercises, patients used parallel bars in the beginning, and crutches or a walker from one week after surgery. By the time of discharge, all patients used a T cane during walking exercises. The length of hospital stay according to the clinical pathway was three weeks. In addition to inpatient rehabilitation, most patients underwent outpatient rehabilitation roughly once a week after discharge and performed activities such as getting up from the floor or ascending and descending stairs according to ability.

### Measurements

We collected subject background information and surgical information, including age, height, body weight, body mass index, disease duration (years), length of hospital stay (days), operative time (minutes), intraoperative blood loss (ml), leg extension (mm), and femoral offset from medical charts. Disease duration was defined as the period from pain onset to surgery, as indicated by patients on a questionnaire [15, 22]. Leg extension and femoral offset were measured by a single researcher using a previously described method [15, 23]. We measured the Japanese Orthopaedic Association hip score, strength of lower extremity muscles (hip abductor and knee extensor) on the operated side, and Timed Up and Go (TUG) test values. All measurements were performed by two physical therapists who had thoroughly practiced measurement procedures. The number of steps was used as an index of postoperative physical activity.

Lower extremity maximal isometric strength was measured using a hand-held dynamometer (μTas F-1; Anima Corp., Tokyo, Japan). We referred to the measuring method proposed by Fukumoto et al 6. For the assessment of hip abductor strength, patients were positioned on a platform in a supine position at a 0° angle, with a sensor pad attached to the distal lateral side of the thigh. For the assessment of knee extensor strength, patients were positioned on a platform in a sitting position at a 90° angle, with a sensor pad attached to the distal front side of the lower leg. After practice, isometric muscle strength during 5 seconds of isometric contraction was measured twice, and the higher value on the hand-held dynamometer was used for analysis. The length of the lever arm (m) was measured from the each of joint to the center of the sensor pad (hip abductor strength used the distance from the greater trochanter to the center of the sensor) [24]. Muscle strength was calculated as torque (Nm).

In the TUG test, the time it took to get up from the chair, go around the mark 3 m ahead, and return to the chair again was measured. The measurement was performed twice at maximum walking speed, and the minimum value was used for analysis. For the measurement of physical activity, a digital pedometer with 3-axis acceleration sensors (TH-400; YAMASA, Tokyo, Japan) was used to count the number of steps (steps/day). The validity and reliability of another device by the same manufacturer (EX-510; YAMAX, Tokyo, Japan), which uses the same algorithm, has been verified previously [25]. Patients were instructed to wear the device on their body for 24 hours a day except when bathing or sleeping (sleep time was set at 8 hours), for one to two months after surgery. Non-wearing time (in hours), if any, was recorded by patient report. If non-wearing time was 4 hours or more, the day was treated as a missing value [26]. The average value for one month was calculated, excluding missing values.

### Statistical analysis

All values are presented as mean (standard deviation) or median (1st quartile to 3rd quartile). The paired t-test or Wilcoxon signed-rank sum test was used to compare Japanese Orthopaedic Association score, TUG value, hip abductor strength, and knee extensor strength 12 months after THA with those measured before and 6 months after THA. The t-test or Mann-Whitney U test was used to compare background factors and TUG values between THA patients and healthy subjects. A general linear model adjusted for body weight was used to compare muscle strength 12 months after THA with that of healthy subjects.

A generalized linear model approach was used to identify factors that determine hip abductor and knee extensor strength 12 months after THA. The generalized linear model is an extension of the linear model, and the probability distribution of any exponential family can be selected for the error structure. In this study, we selected normal distribution and gamma distribution. First, we created a model in which the objective variables were hip abductor and knee extensor strength 12 months after THA, with preoperative muscle strength as the only explanatory variable (Step 1: univariate analysis). Second, we created a model using the forced entry method, with preoperative muscle strength, age, body weight, height, disease duration, physical activity, and leg extension as explanatory variables (Step 2: multivariate analysis). Correlation matrices were calculated for these explanatory variables. Third, a model was created by centering the explanatory variables and adding interaction terms between preoperative muscle strength and age, disease duration, physical activity, and leg extension (Step 3: multivariate analysis with interaction terms). If the interaction term was significant, and residual deviations in Steps 2 and 3 significantly decreased, a simple slope analysis was performed using the method described by Cohen & Cohen [27]. The mean value ± 1SD was adopted as the condition for simple slope analysis. The model with the lowest Akaike’s Information Criterion (AIC), a selection criterion, was used as the final model. The sample size was calculated using the calculation method for the linear model (α = 0.05, detection power = 0.95, effect size = 0.3, 8 input explanatory factors), yielding a required sample size of 84. Since a previous longitudinal study, which assessed patients for up to 12 months after THA, reported a dropout rate of roughly 20-30% [5, 8], we estimated a dropout rate of about 25% and selected 115 subjects for baseline measurements. R version 3.6.2 (R Foundation for Statistical Computing, Vienna, Austria) was used for all statistical analyses.

## Results

**Fig. 1:**
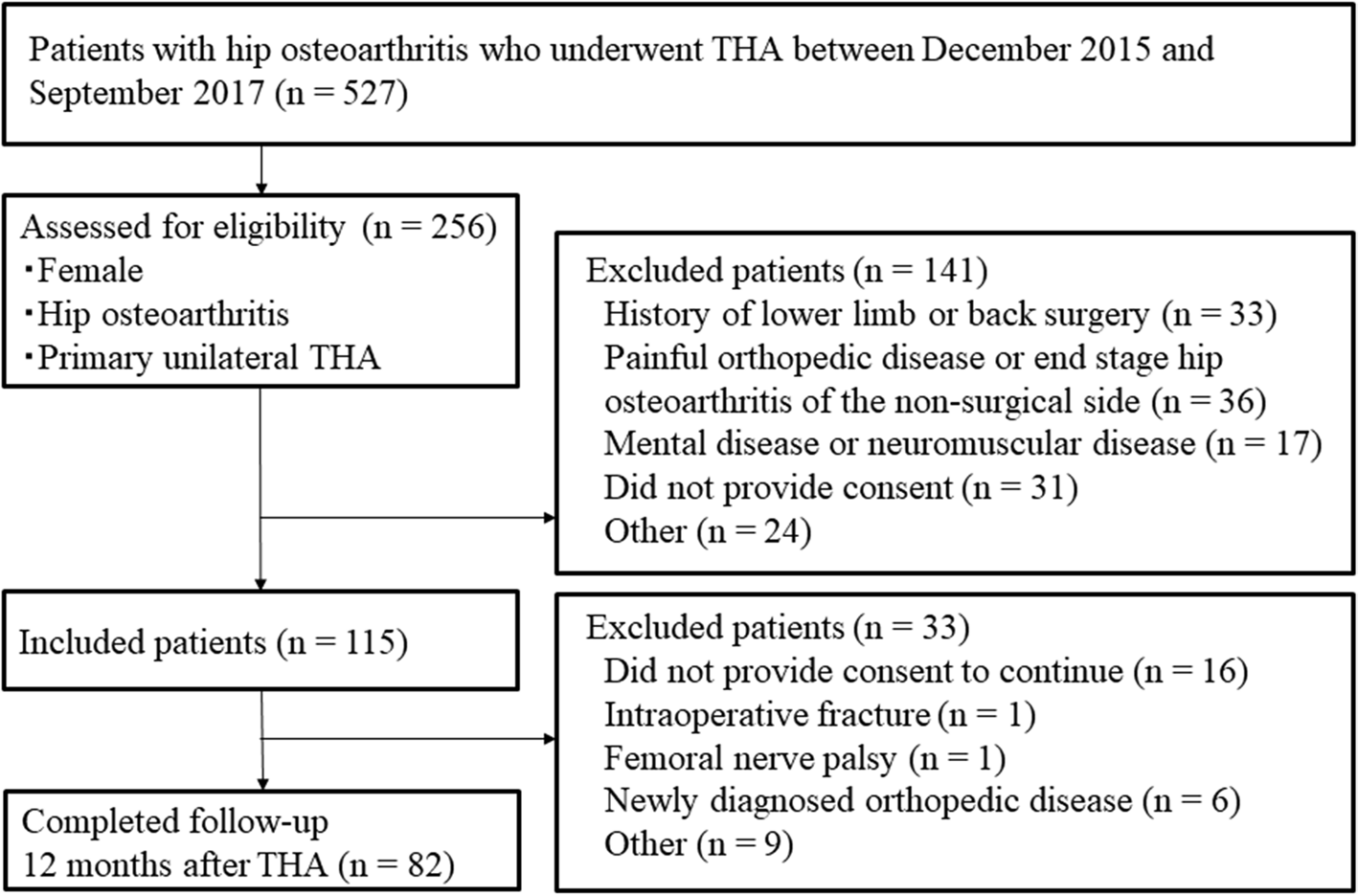
Flow chart of patient selection

Of the 527 patients who underwent unilateral THA at our hospital, 115 female patients completed baseline measurements. Of these, 82 were included in the analysis (Fig. 1) and 33 dropped out (16 did not provide consent to continue, 6 had other orthopedic disorders, 1 had intraoperative fracture, 1 had nerve palsy, and 9 for other reasons). Only one patient had missing data (TUG value at 12 months postoperatively). Table 1 shows background factors for THA patients and healthy subjects, and surgical information and physical activity for THA patients. There were no significant differences in background factors between THA patients and healthy subjects.

**Table 1:** Background factors for THA patients and healthy subjects

	Patient		Control		Patient vs Control	
					p value	95%CI
Background factor						
Age	62.9	(7.9)	66.0	(52.3 to 72.3)	0.771	-3.246 to 4.359
Height (cm)	154.1	(5.2)	155.4	(6.5)	0.156	-3.898 to 0.633
Body weight (kg)	54.8	(49.4 to 59.8)	52.7	(47.2 to 59.3)	0.198	-1.200 to 5.000
BMI	22.8	(21.4 to 25.4)	21.9	(20.4 to 24.1)	0.057	-0.025 to 2.280
Disease duration (years)	19.5	(17.0 to 23.0)				
Length of stay (days)	5.0	(2.6 to 10.0)				
Surgical information						
Operative time (min)	71.0	(62.3 to 81.8)				
Intraoperative blood loss (ml)	205.0	(150.0 to 300.0)				
Leg extension (mm)	8.6	(4.5 to 12.7)				
Femoral offset (mm)	39.6	(4.9)				
Physical activity						
Physical activity (steps/day)	3580.8	(2392.3 to 4971.7)				

*Abbreviations*: *CI* confidence interval

Values are presented as mean (standard deviation) or median (1st quartile to 3rd quartile)

Table 2 shows Japanese Orthopaedic Association hip scores, muscle strength (hip abductor and knee extensor), and TUG values before and 6 and 12 months after THA for THA patients, and muscle strength and TUG values for healthy subjects. Japanese Orthopaedic Association hip score, muscle strength, and TUG value at 12 months after THA were significantly higher relative to before and 6 months after THA (p < 0.001). Hip abductor strength and TUG value at 12 months after THA were significantly lower compared to those of healthy subjects (p < 0.001), but no significant difference was observed for knee extensor strength.

**Table 2:** Japanese Orthopaedic Association hip scores, hip abductor strength, knee extensor strength, and TUG values before (Pre) and 6 months (6M) and 12 months (12M) after THA for THA patients, and muscle strength and TUG value for healthy subjects

								p value (vs 12M)		Control		p value (vs control)	
		Pre		6M		12M		Pre vs	6M vs			6M vs	12M vs
JOA hip score		42.0	(32.0 to 49.0)	75.0	(73.0 to 78.0)	78.0	(76.0 to 80.0)	<.001 ^b^	<.001 ^b^				
Muscle strength (Nm)													
	Hip abductor	40.7	(10.8)	51.1	(12.0)	55.0	(12.3)	<.001 ^a^	<.001 ^a^	69.1	(13.7)	<.001 ^c^	<.001 ^c^
	Knee extensor	53.8	(16.1)	62.3	(16.0)	68.5	(16.4)	<.001 ^a^	<.001 ^a^	71.1	(21.0)	<.001 ^c^	.133 ^c^
TUG value (sec)		8.4	(1.8)	6.9	(1.1)	6.7	(0.9)	<.001 ^a^	<.001 ^a^	5.7	(0.6)	<.001 ^d^	<.001 ^d^

*Abbreviations*: *JOA* Japanese Orthopaedic Association, *TUG* timed up and go

^a^: Paired t-test, ^b^: Wilcoxon signed-rank test, ^c^: general linear model adjusted for body weight, ^d^: two-sample t-test. Values are presented as mean (standard deviation) or median (1st quartile to 3rd quartile). TUG values were compared with missing values excluded (n = 81)

Table 3 shows results of the correlation matrix. Postoperative muscle strength showed a negative correlation with age (p < 0.05) and a positive correlation with height and body weight (p < 0.05), but no significant correlation was found between leg length and disease duration. Preoperative hip abduction muscle strength was significantly correlated with preoperative knee extension muscle strength (p < 0.05), and postoperative hip abduction muscle strength was significantly correlated with postoperative knee extension muscle strength (p < 0.05). Tables 4 and 5 show results of the generalized linear model analysis. For hip abductor strength, AIC of the model was lower when assuming a normal distribution compared to when assuming a gamma distribution. AIC of the multivariate model (Step 2) without an interaction term (607.05) was the lowest, and this was used as the final model for hip abductor strength. On the other hand, for knee extensor strength, AIC of the model was lower when assuming a gamma distribution compared to when assuming a normal distribution. AIC of the multivariate model (Step 3) which included the interaction term between preoperative muscle strength and age (639.8) was the lowest, and this was used as the final model for knee extensor strength. Only the interaction term between preoperative muscle strength and age was found to be a significant factor. Preoperative muscle strength, age, body weight, physical activity, leg extension, and disease duration were extracted as factors that significantly determined hip abductor strength 12 months after THA (Step 2), and preoperative muscle strength, age, body weight, physical activity, disease duration, and the interaction term between preoperative muscle strength and age were extracted as factors that significantly determined knee extensor strength 12 months after THA (p < 0.05) (Step 3).

**Table 3:** Results of the correlation matrix

	Preoperative		Postoperative		Age	Height	Body weight	Physical activity	Leg extension	Disease duration
	Hip abductor	Knee extensor	Hip abductor	Knee extensor						
Preoperative										
Hip abductor		0.550*	0.528*	0.328*	-0.129	0.304*	0.191	0.060	-0.094	-0.036
Knee extensor			0.452*	0.588*	-0.224*	0.323*	0.404*	-0.023	0.064	-0.009
Postoperative										
Hip abductor				0.518*	-0.315*	0.362*	0.365*	0.317*	-0.098	0.037
Knee extensor					-0.383*	0.322*	0.358*	0.148	-0.030	-0.202
Age						-0.330*	-0.127	-0.169	-0.176	-0.043
Height (cm)							0.515*	-0.016	-0.088	0.038
Body weight (kg)								-0.089	0.101	0.025
Physical activity (steps)									0.112	0.185
Leg extension (mm)										0.124
Disease duration (year)										

*: *p* <.05

**Table 4:** kaike’s Information Criterion at each step of the generalized linear model and p-value for each interaction term

	Hip abductor				Knee extensor			
	Gamma		Normal		Gamma		Normal	
	AIC	p value	AIC	p value	AIC	p value	AIC	p value
Step1	623.4		622.1		659.3		661.2	
Step2	610.3		607.05*		642.3		647.3	
Step3								
Age	611.7		609.0	0.829	639.8*	0.046*	648.5	
Physical activity	612.3		608.4	0.462	644.2	0.755	649.3	
Disease duration	610.8		607.8	0.296	643.4	0.376	649.3	
Leg extension	608.9		607.11	0.190	643.1	0.299	648.3	

*Abbreviations*: *AIC* Akaike's Information Criterion

Step 1: Univariate analysis, Step 2: Multivariate analysis, Step 3: Multivariate analysis with interaction terms. *: Model with the smallest Akaike’s Information Criterion

**Table 5:** Results of univariate and multivariate analyses

Hip abductor strength	Step 1			Step 2			Step 3		
	R	p value	95%CI	B	p value	95%CI	B	p value	95%CI
Preoperative muscle strength	6.02×10^-1^	<.001	3.86×10^-1^ to 8.17×10^-1^	4.58×10^-1^	<.001*	-1.14×10^-4^ to -7.74×10^-6^			
Age				-3.10×10^-1^	.033*	-5.90×10^-1^ to -3.04×10^-2^			
Height (cm)				1.08×10^-1^	.672	-3.90×10^-1^ to 6.06×10^-1^			
Body weight (kg)				3.29×10^-1^	.009*	8.71×10^-2^ to 5.71×10^-1^			
Physical activity (steps)				1.50×10^-3^	.005*	4.79×10^-4^ to 2.53×10^-3^			
Leg extension (mm)				-3.18×10^-1^	.017*	5.74×10^-1^ to 6.32×10^-2^			
Disease duration (years)				-2.67×10^-2^	.004*	-3.10×10^-1^ to 2.56×10^-1^			
Knee extensor strength	Step 1			Step 2			Step 3		
	R	p value	95%CI	B	p value	95%CI	B	p value	95%CI
Preoperative muscle strength	-1.12×10^-4^	<.001	-1.14×10^-4^ to -7.74×10^-6^	-8.50×10^-5^	<.001*	-1.14×10^-4^ to -7.74×10^-6^	-8.97×10^-5^	<.001*	-1.24×10^-4^ to -5.44×10^-6^
Age				1.06×10^-4^	.010*	2.78×10^-5^ to 1.83×10^-4^	1.04×10^-4^	.009*	2.87×10^-5^ to 1.80×10^-4^
Height (cm)				-6.21×10^-5^	.348	-1.91×10^-4^ to 6.67×10^-5^	-3.17×10^-5^	.635	-1.61×10^-4^ to 9.86×10^-5^
Body weight (kg)				-5.86×10^-5^	.071	-1.21×10^-4^ to 4.49×10^-6^	-6.51×10^-5^	.043*	-1.26×10^-4^ to -2.78×10^-6^
Physical activity (steps)				-3.31×10^-7^	.019*	-5.97×10^-7^ to -5.77×10^-8^	-2.80×10^-7^	.043*	-5.44×10^-7^ to -1.12×10^-8^
Leg extension (mm)				1.43×10^-5^	.704	-5.78×10^-5^ to 8.94×10^-5^	1.70×10^-5^	.647	-5.39×10^-5^ to 9.06×10^-5^
Disease duration (years)				1.27×10^-4^	.004*	4.49×10^-5^ to 2.11×10^-4^	1.31×10^-4^	.002*	5.08×10^-5^ to 2.14×10^-4^
Age*Preoperative muscle strength							-5.90×10^-6^	.046*	4.49×10^-5^ to 2.11×10^-4^

*Abbreviations*: *C*I confidence interval

Step 2 yielded the final model for hip abductor strength. Step 3 yielded the final model for knee extensor strength. R = regression coefficient, B = partial regression coefficient. *: <0.05

**Fig. 2:**
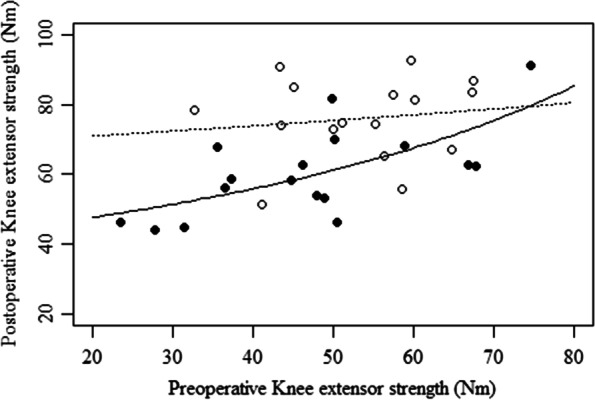
Results of simple slope analysis showing the effect of age on the relationship between preoperative and postoperative knee extensor strength. ●: Elderly (average + 1SD.), ○: Middle-aged (average - 1SD.). A significant regression curve was observed for the elderly group (solid line) (p<0.01), but not for the middle-aged group (dashed line) (p>0.05)

The interaction term between preoperative muscle strength and age significantly reduced the residual deviance of Step 3 compared to Step 2 (Step 2 = 2.18, Step 3 = 2.06; p = 0.042). The variance inflation factor was 10 or less in all models, and multicollinearity was not observed. A simple slope analysis was performed as a subtest (Fig. 2), revealing that regression coefficients for preoperative knee extensor strength and postoperative knee extensor strength (i.e., 12 months after THA) were significant when age was +1 SD (elderly) (p<0.01), but not when age was -1 SD (middle-aged). Middle-aged patients were estimated to have recovered their muscle strength to the same level as the control group (71.7 Nm), regardless of preoperative muscle strength. On the other hand, postoperative muscle strength of the elderly with low preoperative muscle strength was poor, and recovery to the normal value was limited to those having the same muscle strength as the control group prior to THA.

## Discussion

The present prospective observational study is the first to develop a predictive model for hip abductor and knee extensor strength 12 months after THA with an interaction term. Our finding that the interaction term between preoperative muscle strength and age is significant suggests the possibility that the effect of preoperative knee extensor muscle strength on knee extensor muscle strength 12 months after THA depends on age. While middle-aged patients showed good recovery of postoperative muscle strength regardless of preoperative muscle strength, elderly patients with low preoperative muscle strength showed insufficient recovery.

The TUG time 12 months after THA was significantly slower (117%) than that of healthy subjects in this study. This result is similar to that reported in a systematic review [5]. Both hip abductor strength and knee extensor strength 6 months after THA were significantly lower compared to those of healthy subjects (74% and 87%, respectively). Consistent with these findings, a previous study with similar background factors reported a significant reduction in hip abductor and knee extensor strength 6 months after THA (both 75% of healthy subjects) using the same measurement method, suggesting that both muscles remain weak 6 months after THA [6]. In the present study, knee extensor strength, but not hip abductor strength, recovered to the level of healthy subjects 12 months after THA. A previous study, however, reported that hip abductor strength was comparable to that of healthy subjects, whereas knee extensor strength was significantly lower 12 months after THA [8]. This discrepancy may be due to differences in muscle strength measurement methods as well as patient background factors, surgical procedures, and postoperative protocols. As only a few studies have compared lower limb muscle strength between THA patients and healthy subjects, much remains unknown regarding the recovery status of lower limb muscle strength 12 months after THA. Detailed studies, such as multicenter studies, are warranted.

We created statistical models with interaction terms between preoperative muscle strength and age, disease duration, physical activity, and leg extension to clarify factors that determine postoperative muscle strength. Preoperative muscle strength is a strong factor that determines postoperative muscle strength, but there may be interaction effects between preoperative muscle strength and other specific factors. For example, skeletal muscle mass and muscle strength decrease with age [28, 29], and recovery of muscle strength up to 12 months after THA is expected to be poorer in elderly patients than in middle-aged patients. In addition, disuse muscle atrophy due to long-term illness [30, 31], muscle damage due to excessive leg extension, and decreased postoperative physical activity [32] may impede postoperative muscle strength recovery. The inclusion of interaction terms between preoperative muscle strength and specific factors allows for the assessment of whether the partial regression coefficient of preoperative muscle strength is dependent on other factors. If the interaction with a particular factor is significant and increases the partial regression coefficient of preoperative muscle strength, then the expected value of postoperative muscle strength in subjects with low preoperative muscle strength may be even lower. Therefore, including interaction terms not only improves the accuracy of the predictive model, but also helps identify subjects whose postoperative muscle strength is likely to decrease.

The link function of the model for knee extensor strength is an inverse function. When the regression coefficient is negative, postoperative muscle strength increases exponentially as preoperative muscle strength increases. Our analyses revealed that preoperative muscle strength, age, body weight, physical activity, and disease duration were common factors that determine postoperative muscle strength (Step 2 for hip abductor strength and Step 3 for knee extensor strength). Consistent with this, previous studies have reported that preoperative muscle strength, age, body weight, and body mass index are factors related to muscle strength [15, 28, 29], supporting the validity of our models. The interaction between the amount of physical activity and preoperative muscle strength after surgery was not significant, and was not a factor that influenced the effect of preoperative muscle strength. While a number of studies have reported on the causal relationship between muscle strength and physical activity [33, 34], it remains unclear whether maintaining high physical activity 2 months after THA improves muscle strength 12 months after THA.

A significant interaction between preoperative muscle strength and age was observed for knee extensor strength. Muscle strength recovered to almost normal levels in elderly patients with high preoperative muscle strength, and in middle-aged patients regardless of preoperative muscle strength. Knee extensor strength may be difficult to improve in the short term [6], but given that there is no direct effect of osteoarthritis, or intraoperative invasion with the anterior-lateral approach (i.e., there are few structural issues), some improvement is expected in the long term. However, recovery of muscle strength in elderly patients with low preoperative muscle strength was poorer compared to middle-aged patients, and muscle strength 12 months after THA did not reach normal levels. A wide variety of symptoms and conditions are associated with aging, including common disorders such as systemic muscular atrophy and weakness, as well as sarcopenia (less common). These conditions not only reduce skeletal muscle mass but also lower nutritional status and physical activity, making the elderly more prone to disuse syndrome [35]. Elderly patients with preoperative knee extensor weakness are more likely to have generalized muscle weakness, which contributes to increased postoperative muscle weakness and low muscle strength after THA. This may explain why the effect of preoperative muscle strength on postoperative knee extensor strength depends on age. Our findings suggest the need to consider the interaction between preoperative muscle strength and age when predicting postoperative muscle strength. To the best of our knowledge, the present study is the first to examine the effects of interaction terms in models that predict postoperative muscle strength. Our results are unique and important in that they show how preoperative muscle strength can differently affect postoperative muscle strength depending on other factors.

Many patients with hip osteoarthritis reportedly have abductor shortening and atrophy [32, 36]. Hip abductor damage is also observed postoperatively, even with minimally invasive procedures [37]. The abductor muscles often exhibit structural problems, which may impede postoperative muscle strength recovery. Leg extension was extracted as a factor that determines postoperative hip abductor strength, suggesting that abductor degeneration [36] associated with leg shortening and leg extension may inhibit muscle recovery. On the other hand, no significant interaction between preoperative muscle strength and leg extension was observed, indicating that recovery of muscle strength is poor if preoperative muscle strength is low. Structural problems of the abductor muscles are important issues that require further investigation.

There was a strong relationship between preoperative hip abduction muscle strength and preoperative knee extension muscle strength, and between postoperative hip abduction muscle strength and postoperative knee extension muscle strength, in the present study. Previous studies have reported on decreased knee extension muscle strength in patients with hip osteoarthritis and decreased hip abduction muscle strength in patients with knee osteoarthritis [30, 38], suggesting that muscle weakness can occur in regions other than the affected joints. Consistent with this, the stability of the hip joint is reportedly important for exerting knee extensor muscle strength [39]. The process of recovering the strength of muscles in regions other than the affected joints might impact the process of overall postoperative muscle strength recovery. Further studies on this issue are warranted.

In the present study, elderly patients with low preoperative knee extensor strength did not achieve sufficient recovery 12 months after THA, suggesting the need for active training before surgery. Knee extension training includes squatting, an easy, low-risk exercise. The postoperative course is expected to improve if patients are instructed to engage in voluntary training during the waiting period before surgery.

### Limitations

This study has some limitations. First, the number of dropouts was high. In addition, although within the predicted range, nearly 30% of patients were excluded for not completing the long-term survey (i.e., 12 months after THA). Thus, the presence of selection bias cannot be denied. However, when preoperative hip abductor strength and knee extensor strength were compared between dropouts and analyzed patients, no significant difference was observed between the two groups, suggesting that the impact of dropouts may have been minimal. Second, since patients with orthopedic complications other than hip osteoarthritis were excluded, it is possible that only patients with a better than average course of THA were included in our analyses.

## Conclusion

We developed a predictive model for hip abductor and knee extensor strength 12 months after THA with an interaction term. Age, body weight, disease duration, and physical activity were identified as factors that determine strength of both muscles, and leg extension and preoperative muscle strength were extracted as an independent factor that determines hip abductor strength. In the model for knee extensor strength, the effect of preoperative muscle strength on postoperative muscle strength depended on age. While middle-aged patients showed good recovery regardless of preoperative muscle strength, recovery of muscle strength was affected by preoperative muscle strength in elderly patients.

## Data Availability

The datasets used and/or analyzed during the current study are available from the corresponding author on reasonable request.

## Abbreviations

THA: Total hip arthroplasty
TUG: Timed Up and Go test
AIC: Akaike’s Information Criterion
JOA: Japanese Orthopaedic Association
BMI: Body mass index
CI: Confidence interval
